# Natriuretic peptide level at heart failure diagnosis and risk of hospitalisation and death in England 2004–2018

**DOI:** 10.1136/heartjnl-2021-319196

**Published:** 2021-06-28

**Authors:** Clare J Taylor, Sarah L Lay-Flurrie, José M Ordóñez-Mena, Clare R Goyder, Nicholas R Jones, Andrea K Roalfe, FD Richard Hobbs

**Affiliations:** Nuffield Department of Primary Care Health Sciences, University of Oxford, Oxford, UK

**Keywords:** heart failure

## Abstract

**Objective:**

Heart failure (HF) is a malignant condition requiring urgent treatment. Guidelines recommend natriuretic peptide (NP) testing in primary care to prioritise referral for specialist diagnostic assessment. We aimed to assess association of baseline NP with hospitalisation and mortality in people with newly diagnosed HF.

**Methods:**

Population-based cohort study of 40 007 patients in the Clinical Practice Research Datalink in England with a new HF diagnosis (48% men, mean age 78.5 years). We used linked primary and secondary care data between 1 January 2004 and 31 December 2018 to report one-year hospitalisation and 1-year, 5-year and 10-year mortality by NP level.

**Results:**

22 085 (55%) participants were hospitalised in the year following diagnosis. Adjusted odds of HF-related hospitalisation in those with a high NP (NT-proBNP >2000 pg/mL) were twofold greater (OR 2.26 95% CI 1.98 to 2.59) than a moderate NP (NT-proBNP 400–2000 pg/mL). All-cause mortality rates in the high NP group were 27%, 62% and 82% at 1, 5 and 10 years, compared with 19%, 50% and 77%, respectively, in the moderate NP group and, in a competing risks model, risk of HF-related death was 50% higher at each timepoint. Median time between NP test and HF diagnosis was 101 days (IQR 19–581).

**Conclusions:**

High baseline NP is associated with increased HF-related hospitalisation and poor survival. While healthcare systems remain under pressure from the impact of COVID-19, research to test novel strategies to prevent hospitalisation and improve outcomes—such as a mandatory two-week HF diagnosis pathway—is urgently needed.

## Introduction

Heart failure (HF) is a malignant condition affecting around a million people in the UK and has a worse prognosis than most cancers.^1–3^ Survival rates have not, unlike cancer, improved substantially over the last two decades.^4^ A timely diagnosis is key to receiving evidence-based treatments which can both prevent hospitalisation and improve outlook.^5^ Guidelines recommend patients with symptoms suggestive of HF (breathlessness, fatigue, ankle swelling) have a natriuretic peptide (NP) blood test in primary care to determine whether, and how quickly, a specialist diagnostic assessment is required.^6–11^

The National Institute for Health and Care Excellence (NICE) in England recommends echocardiography and cardiology review within 6 weeks if the NP level is raised (BNP ≥100 pg/mL or NT-proBNP ≥400 pg/mL) and within 2 weeks for those with a high NP (BNP >400 pg/mL or NT-proBNP >2000 pg/mL).^7^ The European Society of Cardiology (ESC) has lower thresholds for referral and specialist assessment (BNP ≥35 pg/mL or NT-proBNP ≥125 pg/mL) but does not specify a time interval.^8^ Both guidelines recommend that an alternative diagnosis should be sought for patients with a low NP level.

There is evidence of significant delays in the HF diagnostic pathway with almost 80% of patients first diagnosed on emergency hospital admission.^12^ This is distressing for patients, costly to the health service and usually denotes progression to a later stage of disease. In England, despite NICE guidance, the 2-week target for people with high NP to be seen by a specialist is not compulsory. This differs from cancer pathways where hospitals are mandated to see patients within 2 weeks of primary care referral^13^ and there is evidence that use of urgent ‘2-week wait’ cancer referral pathways is associated with reduced mortality.^14^

The aim of this study was to determine the risk of hospitalisation and death by NP level at time of HF diagnosis using linked routine primary and secondary care data.

## Methods

### Data sources

We conducted a cohort study of men and women between 1 January 2004 to 31 December 2018 using data from the Clinical Practice Research Datalink (CPRD) Gold and Aurum databases, two electronic healthcare records databases drawn from over 1500 general practices in England.^15 16^ Primary care data from CPRD were linked to inpatient Hospital Episodes Statistics (HES) data, Office for National Statistics (ONS) mortality data and Index of Multiple Deprivation (IMD) socioeconomic data.

### Study population

Patients over 45 years of age with a NP test result (in primary care record) and subsequent new diagnosis of HF (in primary or secondary care record) were eligible. Newly diagnosed HF was identified using a comprehensive list of diagnostic codes from the NHS Clinical Terminology Browser and Quality and Outcomes Framework guidelines (online supplemental table S1). Patients were only included if their primary care records were deemed acceptable for research purposes (a CPRD quality measure), eligible for linkage and had been registered at a practice for at least 12 months. Where duplicate patient records appeared in both the Gold and Aurum databases, records were excluded from the Gold database, owing to longer follow-up in the Aurum database.

10.1136/heartjnl-2021-319196.supp1 Supplementary data

### Follow-up and outcomes

Patients entered the cohort on their date of HF diagnosis and exited the cohort on the earliest of the following: date of deregistration with the practice or death, last date of available linked or primary care data, and the end of the study. Outcomes were first all-cause hospital admission within 1 year of diagnosis (drawn from HES data) and all-cause mortality within 10 years (drawn from ONS mortality data). We also explored HF-related hospitalisation and HF-related deaths.

### Study size

Based on our previous work,^9^ this study was powered assuming a 58% increase in deaths within 10 years for those with raised NP compared with those with lower NP levels and assuming <75% of patients would have raised NP. To detect this effect size with 90% power, 5% significance and assuming an overall death rate of 74%,^4^ we required a total of 344 deaths in the study sample.

### Statistical analysis

The association of NP level with mortality and hospital admission was examined using Kaplan-Meier curves and Cox proportional hazards models or logistic regression, respectively. The Fine-Gray competing risks model was used to evaluate HF-related mortality with other causes of death modelled as a single outcome.^15^ Model assumptions were checked visually (residuals plots and log-cumulative hazard plots) and using global test for proportional hazards. Where the proportional hazards assumption was not met, HRs were estimated for separate time windows through time-splitting. Separate analyses were conducted by NP subtype (NT-proBNP and BNP) and the most recent NP value prior to HF diagnosis was used. We examined NP level as a categorical variable according to the NICE categories for referral (NT-proBNP: <400, 400–2000 and >2000 pg/mL; BNP:<100, 100–400 and >400 pg/mL) and as a continuous measure (per 100 pg/mL). NT-proBNP 400–2000 pg/mL and BNP 100–400 pg/mL were used as the reference as the NICE chronic HF guidelines recommend referral through the standard (6 week) route for this group. Possible non-linear relationships were considered using first-order fractional polynomials, retaining the continuous nature of the variables, unlike alternative cut-point approaches. Sensitivity analyses were conducted excluding extreme NP values (BNP >5000 pg/mL and NT-proBNP >10 000 pg/mL). Analyses were partially adjusted first for age and sex and second additionally adjusted for ethnicity (reference=White), IMD quintile (reference=1 least deprived), smoking status (reference=non-smoker, ex-smoker or current smoker), systolic blood pressure, total cholesterol, body mass index (BMI), prior history of angina, myocardial infarction, ischaemic heart disease, diabetes, hypertension, stroke, atrial fibrillation or valve disease and calendar period (2004–2010, 2011–2018). Covariate data were drawn from the primary care record, except in the case of ethnicity (which was drawn from the most recent record in either the primary care record or HES data) and IMD data. Analyses were conducted in complete cases owing to the small amount of missing data present in the study (BMI (4.6%), total cholesterol (7%), others (<1%)). Analysis was carried out using Stata V.16.

### Patient and public involvement

Patients with HF helped to inform the design of this study by sharing their experiences of the pathway to diagnosis. We will work with our PPI group and a national patient-led HF charity, as well as the British Heart Foundation, to disseminate our results.

## Results

In total, 40 247 patients met all inclusion criteria across both databases (see online supplemental figure S2). Of these, 240 died on the same day as the recorded diagnosis of HF and were excluded from further analysis. The characteristics of the remaining 40 007 patients are given in table 1 (48% men and mean age at HF diagnosis of 78.5 years). Baseline NT-proBNP and BNP values were recorded in 27 258 and 13 529 patients, respectively, and values for both were highly skewed (online supplemental figures S2 and S3). Overall, 33.6%, 39.4% and 27.1% of patients had NT-proBNP values of <400, 400–2000 and >2000 pg/mL, respectively, and 26.3%, 42.1% and 31.6% of patients had BNP values of <100, 100–400 and >400 pg/mL, respectively.

**Table 1:** Baseline characteristics of patients overall, and in BNP and NT-proBNP analysis

Variable	Overall		Included in BNP analysis		Included in NT-proBNP analysis	
	N	Mean (SD)/%	N	Mean (SD)/%	N	Mean (SD)/%
Age at diagnosis	40 007	78.5 (9.7)	13 529	78.9 (9.6)	27 258	78.4 (9.7)
NT-proBNP (pg/mL)	27 258	2044 (3733)	780	1069 (1887)	27 258	2044 (3733)
Median (IQR)	27 258	789 (261–2186)	780	388 (148–1155)	27 258	789 (261–2186)
BNP (pg/mL)	13 529	649 (1442)	13 529	649 (1442)	780	481 (1066)
Median (IQR)	13 529	215 (94 to 540)	13 529	215 (94–540)	780	141 (64–372)
Male	19 316	48.3	6480	47.9	13 163	48.3
Smoking status						
Non-smoker	12 118	30.3	4459	33.0	7934	29.1
Current smoker	5868	14.7	1812	13.4	4151	15.2
Ex-smoker	21 930	54.8	7224	53.4	15 116	55.5
Ethnicity						
White	37 755	94.4	12 775	94.4	25 710	94.3
Bangladeshi	47	0.1	16	0.1	31	0.1
Black African	138	0.3	26	0.2	116	0.4
Black Caribbean	310	0.8	80	0.6	236	0.9
Chinese	48	0.1	21	0.2	27	0.1
Indian	503	1.3	189	1.4	331	1.2
Mixed	91	0.2	36	0.3	55	0.2
Other	262	0.7	73	0.5	193	0.7
Other Asian	207	0.5	94	0.7	122	0.4
Other Black	92	0.2	18	0.1	76	0.3
Pakistani	229	0.6	87	0.6	149	0.5
Index of Multiple Deprivation						
Quintile 1 (Most)	8805	22.0	3755	27.8	5203	19.1
Quintile 2	8852	22.1	3119	23.1	5933	21.8
Quintile 3	8347	20.9	2914	21.5	5608	20.6
Quintile 4	7773	19.4	2452	18.1	5484	20.1
Quintile 5 (Least)	6198	15.5	1283	9.5	5003	18.4
Total cholesterol (mmol/L)	37 198	4.6 (1.1)	12 459	4.6 (1.1)	25 452	4.6 (1.1)
Systolic BP (mm Hg)	39 952	137.0 (17.9)	13 512	137.5 (18.0)	27 220	136.8 (17.8)
Diastolic BP (mm Hg)	39 952	75.7 (10.8)	13 512	75.9 (10.9)	27 220	75.6 (10.8)
BMI (kg/m^2^)	38 177	29.2 (6.5)	12 881	29.2 (6.5)	26 042	29.2 (6.5)
Hypertension	28 596	71.5	9806	72.5	19 378	71.1
Diabetes	13 057	32.6	4188	31.0	9158	33.6
Atrial fibrillation	13 760	34.4	4798	35.5	9249	33.9
Angina	6305	15.8	2109	15.6	4340	15.9
Ischaemic heart disease	8396	21.0	2929	21.6	5647	20.7
Myocardial infarction	5218	13.0	1813	13.4	3498	12.8
Stroke	5486	13.7	1794	13.3	3801	13.9
Valve disease	4588	11.5	1617	12.0	3071	11.3
Other CVD	10 942	27.4	3750	27.7	7425	27.2

BP, blood pressure; CVD, cardiovascular disease.

### Time between NP test and HF diagnosis

Overall, the median time between any NP test and HF diagnosis was 101 days (IQR 19–581); 97 days (IQR 19–570) for NT-proBNP and 136 days (IQR 22–729) for BNP. For patients with a moderate NT-proBNP (400–2000 pg/mL) median time from test to diagnosis was 72 days (IQR 16–398) and for a high NT-proBNP (>2000 pg/mL) was 28 days (IQR 7–105). For BNP, the time from test to diagnosis was longer: BNP 100–400 pg/mL: 112 days (IQR 23–631) and BNP >400 pg/ml: 41 days (IQR 9–200).

### NP level and hospitalisation

In total, 22 085 (55.2%) patients were admitted to hospital in the year following HF diagnosis. After adjustment for all confounders, the odds of hospital admission due to any cause for those with an NT-proBNP value of >2000 pg/mL were 20.3% (95% CI 12.8% to 28.3%) higher than the odds for those with NT-proBNP between 400 and 2000 pg/mL (table 2). Those with BNP values of >400 pg/mL also had a 19.2% (95% CI 9.3% to 30.0%) increased odds of hospitalisation (compared with BNP between 100 and 400 pg/mL). In continuous analyses, evidence of non-linearity was inconsistent (square root/linear relationship apparent for NT-proBNP/BNP, respectively) although positive associations were confirmed in continuous linear analysis (table 2) and when removing extreme values (not shown).

**Table 2:** Association of NT-proBNP and BNP with hospital admission at 1 year in people with a new diagnosis of heart failure

	n/N	Unadjusted (N=27 258)	Partially adjusted* (N=27 258)	Fully adjusted† (N=24 434)
		OR (95% CI)	OR (95% CI)	OR (95% CI)
NT-proBNP group (pg/mL)				
<400	4969/9148	1.047 (0.990 to 1.108)	1.029 (0.972 to 1.089)	1.032 (0.971 to 1.097)
400–1999.9 (Reference)	5709/10 738	1.000	1.000	1.000
2000+	4199/7372	1.166 (1.098 to 1.237)	1.169 (1.101 to 1.241)	1.203 (1.128 to 1.283)
NT-proBNP (per 100 pg/mL)	14 877/27 258	1.0013 (1.0006 to 1.0019)	1.0014 (1.0008 to 1.0021)	1.0018 (1.0010 to 1.0025)
	**n/N**	**Unadjusted (N=13 529**)	Partially adjusted* **(N=13 529)**	**Fully adjusted†** (**N=11 913**)
		**OR (95% CI**)	**OR (95% CI**)	**OR (95% CI**)
BNP group (pg/mL)				
<100	2018/3560	1.074 (0.987 to 1.168)	1.055 (0.969 to 1.148)	1.049 (0.956 to 1.150)
100–399.9 (Reference)	3127/5693	1.000	1.000	1.000
400+	2509/4276	1.165 (1.075 to 1.262)	1.161 (1.072 to 1.258)	1.192 (1.093 to 1.300)
BNP (per 100 pg/mL)	7654/13 529	1.0034 (1.0010 to 1.0059)	1.0035 (1.0010 to 1.0060)	1.0037 (1.0010 to 1.0064)

*Adjusted for age and sex.

†Adjusted for age, sex, ethnicity, IMD quintile, smoking status, systolic blood pressure, total cholesterol, body mass index, prior history of angina, myocardial infarction, ischaemic heart disease, diabetes, hypertension, stroke, atrial fibrillation, valve disease and calendar period (2004–2010, 2011–2018). n: number of hospital admissions at 1 year; N: number of persons with a new diagnosis of heart failure.

The most common causes of hospitalisation were diseases of the circulatory system, followed by diseases of the respiratory system (online supplemental table S3). Of those hospitalised, 2069 (9.4%) were admitted for reasons relating to HF. Admissions among those with the highest NT-proBNP levels were more likely to be due to diseases of the circulatory system or HF, compared with admissions among other groups (p<0.0001 for both). The same was true for patients with the highest BNP values (above 400 pg/mL compared with below, p<0.001 for both).

### NP level and mortality

**Figure 1:**
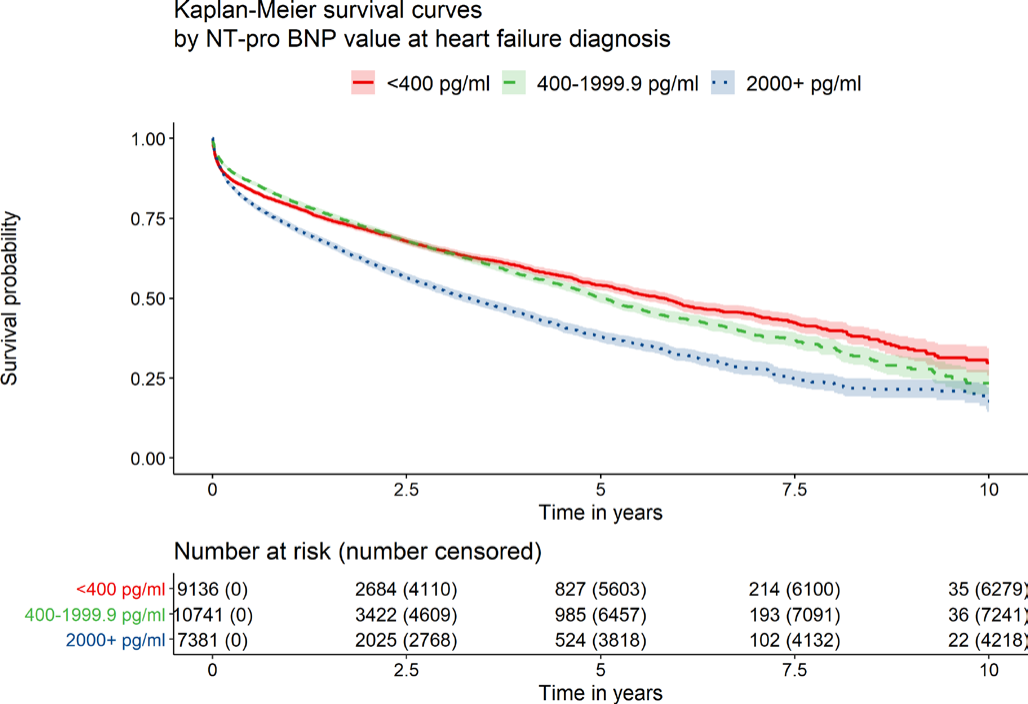
Kaplan-Meier survival curves by NT-proBNP level at heart failure diagnosis.

We observed 14 284 deaths over 10 years of follow-up (79 664 person-years of follow-up). HF was listed as a primary or contributing cause in 769 (5.4%) and 5348 (37.4%) of deaths, respectively. Median survival time from diagnosis was 4.67 years (95% CI 4.55 to 4.77) and varied by NT-proBNP value (figure 1 and online supplemental figure S4). Risk of death was highest in those with NT-proBNP >2000 pg/mL in short- and long-term follow-up (online supplemental table S4). Mortality rates in the >2000 pg/mL group were 27%, 62% and 82% at 1, 5 and 10 years, compared with 19%, 50% and 77%, respectively, in the 400–2000 pg/mL group.

There was evidence that the association between NT-proBNP and mortality differed in the early compared with later years of follow-up (global test for proportional hazards, p<0.001) and separate estimates of association were derived for years 0–2 and 2–10 of follow-up using an interaction term with time. In unadjusted and adjusted regression analyses, those with a baseline NT-proBNP value of >2000 pg/mL were at consistently higher risk of death (table 3). In fully adjusted analysis, compared with those with NT-proBNP values between 400 and 2000 pg/mL, those with a value of >2000 pg/mL were at 41.4% (95% CI 33.0% to 50.3%) greater risk of death in the first 2 years of follow-up and at 34.3% (95% CI 21.3% to 48.7%) greater risk in years two to ten. Evidence of a difference in mortality risk between those with NT-proBNP values below 400 pg/mL and those with NT-proBNP values between 400 and 2000 pg/mL was not consistent. In analysis considering NT-proBNP as a continuous measure, the association between higher NT-proBNP values and higher mortality was confirmed (online supplemental table S5). There was no consistent evidence of non-linearity with the linear term retained in fractional polynomial analyses when fitting both partially and fully adjusted models over 1, 5 and 10 years of follow-up. Comparing these findings with those in table 3, this suggests that analyses of continuous data was driven by events occurring after 2 years. Similar results were observed after excluding extreme values (data not shown). Considering NT-proBNP in a greater number of categories (online supplemental table S6) suggested that the risk of death began to increase at values of 800 pg/mL or higher. There was no increase in risk of death seen between the lower limits for referral in the ESC and NICE guidelines—NT-proBNP <125 pg/mL (ESC) vs <400 pg/mL (NICE).

**Table 3:** Association of NT-proBNP with all-cause mortality at 1, 5 and 10 years of follow-up (estimated with Cox proportional hazards models with a time-split at 2 years)

Total follow-up	NT-proBNP group (pg/ml)	n/PY	Unadjusted (N=27 258)	Partially adjusted* (N=27 258)	Fully adjusted† (N=24 434)
			HR (95% CI)	HR (95% CI)	HR (95% CI)
1 year	<400	1761/6584	1.132 (1.061 to 1.208)	1.283 (1.202 to 1.370)	1.235 (1.150 to 1.327)
	400–1999.9 (Reference)	1890/8086	1.000	1.000	1.000
	2000+	1846/5208	1.484 (1.392 to 1.583)	1.404 (1.317 to 1.497)	1.404 (1.310 to 1.506)
5 years	Years 0–2:				
	<400	2198/10 776	1.082 (1.021 to 1.146)	1.240 (1.171 to 1.314)	1.214 (1.140 to 1.293)
	400–1999.9 (Reference)	2483/13 312	1.000	1.000	1.000
	2000+	2395/8449	1.485 (1.404 to 1.571)	1.403 (1.326 to 1.484)	1.414 (1.330 to 1.508)
	Years 2–5:				
	<400	515/5569	0.793 (0.710 to 0.885)	0.900 (0.806 to 1.006)	0.891 (0.791 to 1.003)
	400–1999.9 (Reference)	812/6966	1.000	1.000	1.000
	2000+	641/4018	1.369 (1.235 to 1.519)	1.350 (1.217 to 1.497)	1.383 (1.237 to 1.548)
10 years	Years 0–2:				
	<400	2198/10 776	1.082 (1.021 to 1.146)	1.242 (1.172 to 1.316)	1.218 (1.144 to 1.297)
	400–1999.9 (Reference)	2483/13 312	1.000	1.000	1.000
	2000+	2395/8449	1.485 (1.404 to 1.571)	1.402 (1.325 to 1.483)	1.414 (1.330 to 1.503)
	Years 2–10:				
	<400	666/6978	0.793 (0.719 to 0.875)	0.899 (0.815 to 0.992)	0.890 (0.801 to 0.988)
	400–1999.9 (Reference)	1013/8450	1.000	1.000	1.000
	2000+	765/4832	1.322 (1.203 to 1.452)	1.314 (1.196 to 1.443)	1.343 (1.213 to 1.487)

*Adjusted for age and sex.

†Adjusted for age, sex, ethnicity, IMD quintile, smoking status, systolic blood pressure, total cholesterol, body mass index, prior history of angina, myocardial infarction, ischaemic heart disease, diabetes, hypertension, stroke, atrial fibrillation, valve disease and calendar period (2004–2010, 2011–2018)

n, number of deaths; PY, number of person-years.

The primary cause of death was more likely to be attributed to diseases of the circulatory system (49.4% of deaths), and specifically HF (4.8%), in those with NT-proBNP >2000 pg/mL (Table S) compared with those with lower NP values (36.0% and 4.8%, respectively, p<0.001 for both). Consequently, fewer deaths were attributed to disease of the respiratory system or neoplasms in the high NT-proBNP group.

**Figure 2:**
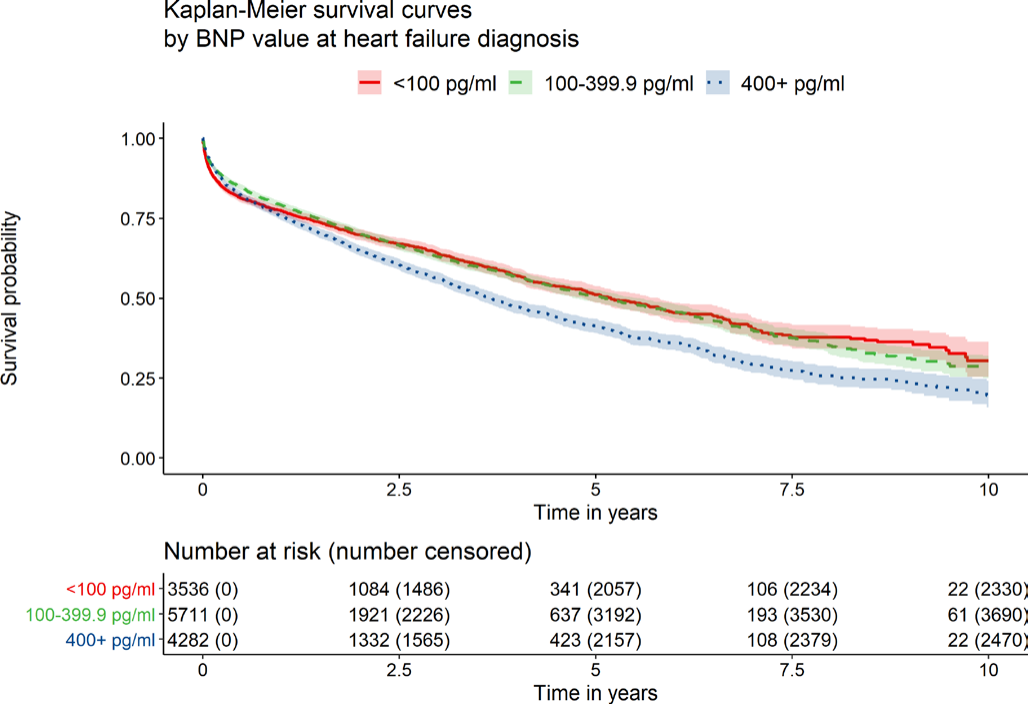
Kaplan-Meier survival curves by BNP level at heart failure diagnosis.

A similar association was observed between BNP values and mortality, with those with BNP >400 pg/mL being at highest risk of death (unadjusted analyses, figure 2 and online supplemental table S8). As with NT-proBNP, the relationship between BNP level and risk of death appeared to violate the proportional hazards assumption (global test for proportional hazards, p<0.001) and separate HRs were estimated at two follow-up periods—years 0–2 and 2–10. In adjusted analyses, those with BNP values>400 pg/mL were at 15.4% (95% CI 6.3% to 25.4%) greater risk of death in years 0–2 of follow-up (compared with those with BNP 100–400 pg/mL) and at 32.4% (95% CI 16.4% to 50.6%) greater risk in years 2 to 10 (online supplemental table S9). This relationship in later years was confirmed in analysis considering BNP as a continuous variable (Table S10). As with NT-proBNP, compared with those with BNP <100 pg/mL those with high BNP were more likely to die from diseases of the circulatory system (p<0.001) and HF (p=0.006, online supplemental table S7).

### HF-related hospitalisation and mortality

After adjustment for all confounders, the odds of admission due to HF for those with an NT-proBNP value of >2000 pg/mL were more than twofold higher (OR 2.26 95% CI 1.98 to 2.59) than for those with NT-proBNP between 400 and 2000 pg/mL whereas a low NT-proBNP level <400 pg/mL was associated with a 29% lower risk (OR 0.71 95% CI 0.61 to 0.84) of HF-related hospitalisation (online supplemental table S11). In a competing risks model, risk of death due to HF was constant over time with a 50% higher risk of HF-related death at 1, 5 and 10 years associated with NT-proBNP >2000 pg/mL compared with NT-proBNP between 400 and 2000 pg/mL (online supplemental table S12).

## Discussion

This large, contemporary community-based study has shown that high NP at diagnosis (NT-proBNP >2000 pg/mL) is associated with more than a twofold increased risk of HF-related hospitalisation in the first year, and higher risk of death in the short and long-term, compared with moderate NP levels (NT-proBNP 400–2000 pg/mL). There was no increase in risk of all-cause hospitalisation or death seen between the lower limits for referral in the ESC and NICE guidelines. Time from NP test to HF diagnosis was outside guideline recommended limits.

### Strengths and weaknesses of the study

We used national primary care and registry data known to be representative of the UK population.^16 17^ Patients with HF were included on the basis of a record of HF in either their primary care or hospital record, as previous studies have shown that HF diagnoses are likely to be missed using a single source of data alone.^18^ Ejection fraction data were only available in 836 patients (2.1%) and so could not be included in our analyses. NP levels can be influenced by factors such as renal function, concomitant medication and comorbidities (eg, atrial fibrillation).^19^ However, we decided not to report these subgroups separately as the NICE guideline referral threshold recommendations are based on NP result alone. Cause of death was determined by the information provided on the death certificate and, while certification can be inaccurate, this is likely to be the most reliable data source available in the UK.

Although our results were similar when adjusting for age and sex alone and a wider range of possible confounders, residual confounding cannot be ruled out due to the routine nature of our data sources. Lack of racial diversity is also a limitation as, although race was adjusted for, 94.4% of the cohort was white. Although we restricted our analyses to complete cases, missing data were limited and unlikely to meet the missing at random assumption necessary to implement methods such as multiple imputation.

### Comparisons with previous studies

Several studies have demonstrated associations between higher NP and increased mortality in the general population. The EchoCardiographic Heart of England Screening study showed that a NT-proBNP >150 pg/mL was associated with a 58% increase in risk of death within 10 years.^9^ A substudy of the MONICA cohort, which included adults with raised NP levels, also found mortality risk was doubled for those with BNP >17.9 pg/mL.^20^ More recently, a study of data from two hospital trusts in the South of England showed that those referred to specialist HF clinics via the NICE 2-week pathway (NT-proBNP >2000 pg/mL) were at greater risk of hospitalisation and mortality at 1 year than those referred via the 6-week pathway (NT-proBNP 400–2000 pg/mL).^21^ Studies in patients with established HF also demonstrate increased risk of hospitalisation and mortality. A recent Swedish study of hospitalised patients,^22^ and an older UK study of patients in the community,^9^ both demonstrated increased risks of hospitalisation and mortality with raised NT-proBNP. A 2015 systematic review also showed an increased risk of death associated with raised BNP.^23^ Our analyses support these direct associations but generally indicate associations of a smaller magnitude. This study differs from those previously in terms of the included population (primary care) and represents a more recent calendar time period of follow-up. We also included different adjustment variables in our models.

### Implications for research and practice

We have shown that those with NP levels above the current guideline-based threshold for 2-week referral have high rates of hospitalisation and poor survival. In cancer, evidence suggests that use of rapid referral schemes is associated with reduced mortality, primarily through earlier detection.^14^ The provision of diagnostic services for HF varies considerably and while some areas in England have an urgent referral pathway for patients with a high NP, national compulsory 2-week targets like those seen in cancer services are not currently in place. The Cancer Plan in 2000 shone a light on delayed diagnosis and poor outcomes in cancer and the same policy driven approach may be needed in HF.^24 25^ Mandating the commissioning of similar rapid diagnostic and early treatment pathways in HF could help to improve outcomes.

The COVID-19 pandemic has placed immense strain on healthcare services globally and there is evidence of excess mortality due to cardiovascular disease.^26^ NP-guided referral could be a useful tool for prioritising patients with symptoms of HF in hospitals struggling to maintain usual services in peak surges during the pandemic. It may also be important as healthcare systems seek to ‘catch-up’ on care for patients waiting to be seen for diagnosis by a specialist or who have developed HF as a complication of delayed treatment.^27–29^

## Conclusion

A high NP at HF diagnosis is associated with increased risk of hospitalisation and death in the short and longer term. At a time when healthcare systems are under strain, rapid HF diagnostic and treatment pathways, like those found in cancer services, may prevent unnecessary hospital admission and potentially improve survival.

**Key messages**

**What is already known on this subject?**

- Heart failure (HF) is a malignant condition and natriuretic peptide (NP) testing in primary care is used to prioritise referral for specialist diagnostic assessment.
- The National Institute for Health and Care Excellence recommend that patients with a high NP level (NT-proBNP >2000 pg/mL) should be seen within 2 weeks of referral.

**What might this study add?**

- A NT-proBNP level >2000 pg/mL is associated with a twofold increased risk of HF-related hospitalisation in the year following diagnosis and 41.4% increased mortality in the first 2 years.
- Time from NP test to HF diagnosis was outside guideline recommended limits.

**How might this impact on clinical practice?**

- Patients with a high NP level require urgent diagnostic assessment and treatment initiation.
- Healthcare system changes, such as the introduction of national compulsory 2-week targets like those seen in cancer services, may be needed to improve outcomes for patients and reduce pressure on hospitals.

## Data Availability

Data may be obtained from a third party and are not publicly available. Data for this study was obtained on licence from CPRD and cannot be shared. Equivalent data can be obtained directly from CPRD with relevant ISAC approval.
