# Supplementary material for: Natriuretic peptide level at heart failure diagnosis and risk of hospitalisation and death in England 2004–2018
Source: Heart. 2021 Jun 28;108(7):543–9. doi: 10.1136/heartjnl-2021-319196 (PMC8921592; doi:10.1136/heartjnl-2021-319196)
Supplement: Supplementary data [file heartjnl-2021-319196supp001.pdf]

SUPPLEMENTARY FILE

Tables

Table S1 Clinical codes used to identify heart failure

Medcode	Read Code	Read Term
398	G580.00	Congestive heart failure
884	G581.00	Left ventricular failure
2062	G58..00	Heart failure
2906	G580.11	Congestive cardiac failure
12627	9N0k.00	Seen in heart failure clinic
4024	G58z.00	Heart failure NOS
19002	9N2p.00	Seen by community heart failure nurse
17851	8HBE.00	Heart failure follow-up
12366	662T.00	Congestive heart failure monitoring
30779	662W.00	Heart failure annual review
1223	G58..11	Cardiac failure
9913	1O1..00	Heart failure confirmed
13189	662g.00	New York Heart Association classification - class II
5942	G581.13	Impaired left ventricular function
72965	9Or3.00	Heart failure monitoring first letter
18853	662f.00	New York Heart Association classification - class I
19066	662h.00	New York Heart Association classification - class III
32911	9Or..00	Heart failure monitoring administration
19380	9Or0.00	Heart failure review completed
46672	388D.00	New York Heart Assoc classification heart failure symptoms
15058	14A6.00	H/O: heart failure
70619	8HHz.00	Referral to heart failure exercise programme
5255	G581000	Acute left ventricular failure
83502	662p.00	Heart failure 6 month review
32671	G580100	Chronic congestive heart failure
32945	8CL3.00	Heart failure care plan discussed with patient
9524	G580.14	Biventricular failure
10079	G580.12	Right heart failure
72386	9Or4.00	Heart failure monitoring second letter
60099	67D4.00	Heart failure information given to patient
27884	G580200	Decompensated cardiac failure
26115	8HHb.00	Referral to heart failure nurse
103732	8CMK.00	Has heart failure management plan
17278	G58z.12	Cardiac failure NOS
23707	G580000	Acute congestive heart failure
95835	679X.00	Heart failure education
27964	G582.00	Acute heart failure
10154	G580.13	Right ventricular failure
48897	8HTL.00	Referral to heart failure clinic
26242	ZRad.00	New York Heart Assoc classification heart failure symptoms
23481	G581.11	Asthma - cardiac
110101	8I98.00	Heart failure rehabilitation programme not available
51214	662i.00	New York Heart Association classification - class IV
89650	9Or5.00	Heart failure monitoring third letter
106894	8IE1.00	Referral to heart failure exercise programme declined
101138	G583.00	Heart failure with normal ejection fraction
43618	G581.12	Pulmonary oedema - acute
32898	8H2S.00	Admit heart failure emergency
90193	9Or1.00	Heart failure monitoring telephone invite
83481	9N4w.00	Did not attend heart failure clinic
11424	G580300	Compensated cardiac failure
104275	G584.00	Right ventricular failure
106897	G583.12	Heart failure with preserved ejection fraction
22262	G1yz100	Rheumatic left ventricular failure

Taylor CJ, *et al. Heart* 2022; 108:543–549. doi: 10.1136/heartjnl-2021-319196

90192	9Or2.00	Heart failure monitoring verbal invite
12590	G58z.11	Weak heart
46912	14AM.00	H/O: Heart failure in last year
106680	8HTL000	Referral to rapid access heart failure clinic
102585	8HgD.00	Discharge from heart failure nurse service
106198	661M500	Heart failure self-management plan agreed
94870	G580400	Congestive heart failure due to valvular disease
106008	8CMW800	Heart failure clinical pathway
101137	G583.11	HFNEF - heart failure with normal ejection fraction
95021	9N4s.00	Did not attend practice nurse heart failure clinic
21837	G232.00	Hypertensive heart&renal dis wth (congestive) heart failure
105002	679W100	Education about deteriorating heart failure
69062	9N6T.00	Referred by heart failure nurse specialist
71235	8Hk0.00	Referred to heart failure education group
105542	8CeC.00	Preferred place of care for next exacerbation heart failure
107981	8IE0.00	Referral to heart failure education group declined
91288	8Hg8.00	Discharge from practice nurse heart failure clinic
66306	SP11111	Heart failure as a complication of care
111428	2JZ..00	On optimal heart failure therapy
96799	G5y4z00	Post cardiac operation heart failure NOS

Table S2: Association of NT-pro BNP (6 categories) with hospital admission at 1 year

	Unadjusted (N=27,258)			Partially adjusted[1] (N = 27,258)			Fully adjusted[2] (N = 24,434)		
**NT-pro BNP group (Reference =125-400 pg/ml)**	**OR**	**95% CI**		**OR**	**95% CI**		**OR**	**95% CI**	
**<125**	1.043	0.958	1.125	0.997	0.915	1.086	0.984	0.899	1.077
**400-800**	0.930	0.860	1.006	0.932	0.861	1.007	0.923	0.850	1.003
**800-1200**	1.009	0.921	1.105	1.009	0.921	1.105	1.008	0.914	1.111
**1200-2000**	0.994	0.913	1.083	0.996	0.914	1.085	0.986	0.899	1.082
**2000+**	1.131	1.055	1.212	1.135	1.058	1.218	1.161	1.075	1.254

1. Adjusted for age and sex.
2. Adjusted for age, sex, ethnicity, IMD quintile, smoking status, systolic blood pressure, total cholesterol, body mass index, prior history of angina, myocardial infarction, ischaemic heart disease, diabetes, hypertension, stroke, atrial fibrillation, valve disease and calendar period (2004-2010, 2011-2018).

Table S3: Proportion of hospitalisations attributed to major ICD10 headings for primary cause, overall and by NT-pro BNP/ BNP category

**Cause**	**Overall N=22,085**	**NT-pro BNP <2000 pg/ml N= 10,678**	**NT-pro BNP 2000+ pg/ml N= 4,199**	**BNP 400 pg/ml N= 5,145**	**BNP 400+ pg/ml N=2,509**
**Certain infectious and parasitic diseases**	2.4%	2.6%	2.2%	2.5%	1.7%
**Congenital malformations, deformations and chromosomal abnormalities**	0.1%	0.1%	0.1%	0.1%	0.0%
**Diseases of the blood and blood-forming organs**	3.1%	3.2%	3.4%	2.8%	3.0%
**Diseases of the circulatory system**	31.4%	27.9%	40.1%	28.4%	37.5%
**Heart failure**	9.4%	7.1%	15.4%	7.4%	13.4%
**Diseases of the digestive system**	7.6%	8.4%	6.4%	7.9%	6.3%
**Diseases of the ear and mastoid process**	0.2%	0.2%	0.1%	0.2%	0.1%
**Diseases of the eye and adnexa**	4.2%	4.2%	3.8%	4.6%	4.3%
**Diseases of the genitourinary system**	5.2%	5.1%	5.9%	5.1%	4.6%
**Diseases of the musculoskeletal system and connective tissue**	4.5%	5.3%	2.8%	4.4%	3.6%
**Diseases of the nervous system**	1.3%	1.4%	1.0%	1.6%	1.0%
**Diseases of the respiratory system**	11.8%	12.7%	10.0%	12.5%	9.5%
**Diseases of the skin and subcutaneous tissue**	2.0%	2.0%	1.4%	2.3%	2.2%
**Endocrine, nutritional and metabolic diseases**	2.2%	2.1%	2.7%	2.0%	2.6%
**Factors influencing health status and contact with health services**	1.6%	1.5%	1.4%	1.9%	1.4%
**Injury, poisoning and certain other consequences of external causes**	4.7%	4.7%	5.0%	4.4%	4.8%
**Mental and behavioural disorders**	0.7%	0.7%	0.6%	0.6%	0.8%
**Neoplasms**	5.9%	6.3%	4.5%	6.2%	5.4%
**Symptoms, signs and abnormal clinical and laboratory findings, not elsewhere classified**	11.2%	11.7%	8.7%	12.6%	10.9%

Taylor CJ, *et al. Heart* 2022; 108:543–549. doi: 10.1136/heartjnl-2021-319196

Table S4: Unadjusted all-cause mortality risk by NT-pro BNP group at 1, 5 and 10 years of follow-up

	Follow-up time								
	1 year			5 years			10 years		
Baseline NT-pro BNP value pg/ml	%	95% CI		%	95% CI		%	95% CI	
<400	21.0	20.2	21.9	46.0	44.4	47.6	70.2	65.9	74.5
400-1999.9	19.3	18.6	20.1	49.9	48.4	51.4	76.5	72.6	80.2
2000+	27.3	26.3	28.4	62.1	60.4	63.9	82.2	78.2	85.8

Table S5: Association of NT-pro BNP with all-cause mortality with NT-pro BNP modelled as a continuous, linear exposure

		Unadjusted (N=27,258)			Partially adjusted[1] (N = 27,258)			Fully adjusted[2] (N = 24,434)		
Total follow-up	NT-pro BNP value (Per 100 pg/ml)	HR	95% CI		HR	95% CI		HR	95% CI	
1 year		1.0049	1.0044	1.0054	1.0042	1.0037	1.0047	1.0039	1.0033	1.0045
5 years	Years 0 to 2	1.0048	1.0044	1.0053	1.0041	1.0036	1.0045	1.0039	1.0034	1.0044
	Years 2 to 5	1.0048	1.0038	1.0058	1.0042	1.0032	1.0053	1.0047	1.0036	1.0058
10 years	Years 0 to 2	1.0048	1.0044	1.0053	1.0041	1.0036	1.0045	1.0039	1.0034	1.0044
	Years 2 to 10	1.0043	1.0034	1.0053	1.0038	1.0029	1.0047	1.0045	1.0034	1.0055

1. Adjusted for age and sex
2. Adjusted for age, sex, ethnicity, IMD quintile, smoking status, systolic blood pressure, total cholesterol, body mass index, prior history of angina, myocardial infarction, ischaemic heart disease, diabetes, hypertension, stroke, atrial fibrillation, valve disease and calendar period (2004-2010, 2011-2018)

Taylor CJ, *et al. Heart* 2022; 108:543–549. doi: 10.1136/heartjnl-2021-319196

Table S6: Association of NT-pro BNP (6 categories) with all-cause mortality at 1, 5 and 10 years of follow-up (estimated with Cox proportional hazards models with a time-split at 2 years).

		Unadjusted (N=27,258)			Partially adjusted[1] (N = 27,258)			Fully adjusted[2] (N = 24,434)		
**Total follow-up**	**NT-pro BNP value (Reference 125-400 pg/ml)**	**HR**	**95% CI**		**HR**	**95% CI**		**HR**	**95% CI**	
**1 year**	**<125**	0.903	0.819	0.995	1.067	0.968	1.177	1.056	0.950	1.173
	**400-800**	0.823	0.752	0.902	0.784	0.715	0.859	0.779	0.706	0.860
	**800-1200**	0.832	0.748	0.925	0.784	0.705	0.872	0.818	0.729	0.918
	**1200-2000**	0.904	0.819	0.996	0.826	0.749	0.911	0.898	0.808	0.999
	**2000+**	1.262	1.172	1.360	1.119	1.039	1.206	1.161	1.069	1.262
**5 years**	**Years 0 to 2:**									
	**<125**	0.892	0.818	0.973	1.063	0.974	1.161	1.042	0.949	1.145
	**400-800**	0.859	0.793	0.931	0.812	0.749	0.880	0.807	0.740	0.880
	**800-1200**	0.869	0.792	0.954	0.813	0.741	0.893	0.829	0.750	0.917
	**1200-2000**	0.938	0.860	1.022	0.848	0.778	0.924	0.885	0.806	0.972
	**2000+**	1.316	1.232	1.406	1.155	1.082	1.235	1.185	1.102	1.274
	**Years 2 to 5:**									
	**<125**	0.904	0.755	1.082	1.062	0.887	1.271	1.095	0.905	1.325
	**400-800**	1.072	0.918	1.253	1.017	0.870	1.188	1.027	0.869	1.213
	**800-1200**	1.277	1.077	1.513	1.204	1.016	1.427	1.237	1.031	1.485
	**1200-2000**	1.360	1.160	1.595	1.234	1.052	1.447	1.288	1.084	1.530
	**2000+**	1.663	1.456	1.900	1.531	1.341	1.749	1.609	1.393	1.859
**10 years**	**Years 0 to 2:**									
	**<125**	0.892	0.818	0.973	1.064	0.975	1.162	1.045	0.951	1.147
	**400-800**	0.859	0.793	0.931	0.811	0.748	0.879	0.806	0.740	0.879
	**800-1200**	0.869	0.792	0.954	0.812	0.740	0.892	0.827	0.748	0.914
	**1200-2000**	0.938	0.860	1.022	0.846	0.776	0.923	0.881	0.802	0.967
	**2000+**	1.316	1.232	1.406	1.153	1.079	1.232	1.181	1.098	1.270
	**Years 2 to 10:**									
	**<125**	0.928	0.792	1.086	1.077	0.919	1.261	1.110	0.938	1.312
	**400-800**	1.071	0.932	1.229	1.012	0.881	1.162	1.021	0.881	1.184
	**800-1200**	1.324	1.138	1.539	1.242	1.068	1.444	1.274	1.084	1.500
	**1200-2000**	1.362	1.182	1.569	1.234	1.071	1.422	1.289	1.106	1.502
	**2000+**	1.621	1.439	1.825	1.500	1.332	1.690	1.572	1.381	1.788

1. Adjusted for age and sex.
2. Adjusted for age, sex, ethnicity, IMD quintile, smoking status, systolic blood pressure, total cholesterol, body mass index, prior history of angina, myocardial infarction, ischaemic heart disease, diabetes, hypertension, stroke, atrial fibrillation, valve disease and calendar period (2004-2010, 2011-2018)

Taylor CJ, *et al. Heart* 2022; 108:543–549. doi: 10.1136/heartjnl-2021-319196

Table S7: Proportion of deaths attributed to major ICD10 headings for primary cause, overall and by NT-pro BNP/ BNP category

Cause	Overall N=14,248	NT-pro BNP <2000 pg/ml N= 6,360	NT-pro BNP 2000+ pg/ml N= 3,160	BNP 400 pg/ml N= 3,228	BNP 400+ pg/ml N=1,811
Certain infectious and parasitic diseases	1.3%	1.5%	1.3%	1.2%	0.8%
Congenital malformations, deformations and chromosomal abnormalities	0.1%	0.1%	0.1%	0.1%	0.1%
Diseases of the blood and blood-forming organs	0.3%	0.3%	0.2%	0.6%	0.2%
Diseases of the circulatory system	40.4%	36.0%	49.4%	35.5%	49.1%
Heart failure	5.4%	4.8%	7.3%	4.5%	6.3%
Diseases of the digestive system	4.7%	5.2%	3.5%	4.6%	4.6%
Diseases of the eye and adnexa	0.0%	0.0%	0.0%	0.0%	0.0%
Diseases of the genitourinary system	2.7%	2.2%	3.6%	2.6%	3.1%
Diseases of the musculoskeletal system and connective tissue	0.9%	0.8%	0.8%	1.1%	1.2%
Diseases of the nervous system	1.9%	2.2%	1.2%	2.4%	1.3%
Diseases of the respiratory system	22.6%	24.4%	18.3%	25.1%	19.8%
Diseases of the skin and subcutaneous tissue	0.9%	1.0%	0.6%	0.8%	1.1%
Endocrine, nutritional and metabolic diseases	2.1%	2.0%	2.2%	2.1%	2.2%
External causes of morbidity and mortality	2.1%	2.2%	2.1%	2.2%	1.7%
Mental and behavioural disorders	3.1%	3.2%	2.9%	3.2%	2.9%
Neoplasms	15.7%	17.8%	12.3%	17.5%	11.2%
Symptoms, signs and abnormal clinical and laboratory findings, not elsewhere classified	1.0%	0.9%	1.3%	0.9%	0.8%
Unknown	0.1%	0.1%	0.1%	0.0%	0.0%

Table S8: Unadjusted all-cause mortality risk by BNP group at 1, 5 and 10 years of follow-up

	Follow-up time								
	1 year			5 years			10 years		
Baseline BNP value pg/ml	%	95% CI		%	95% CI		%	95% CI	
<100	22.6	21.2	24.1	48.8	46.3	51.4	69.5	64.0	74.8
100-399.9	20.9	19.8	22.0	49.4	47.4	51.3	71.6	68.1	75.1
400+	24.3	23.0	25.7	58.8	56.6	60.9	80.7	76.3	84.7

Taylor CJ, *et al. Heart* 2022; 108:543–549. doi: 10.1136/heartjnl-2021-319196

Table S9: Association of BNP with all-cause mortality at 1, 5 and 10 years of follow-up (estimated with Cox proportional hazards models with a time-split at 2 years).

		Unadjusted (N=13,529)			Partially adjusted[1] (N = 13,529)			Fully adjusted[2] (N = 11,913)		
Total follow-up	BNP value (Reference = 100-399.9 pg/ml)	HR	95% CI		HR	95% CI		HR	95% CI	
1 year	<100	1.132	1.031	1.242	1.246	1.136	1.368	1.224	1.103	1.358
	400+	1.171	1.074	1.277	1.143	1.048	1.246	1.126	1.024	1.238
5 years	Years 0 to 2:									
	<100	1.071	0.986	1.163	1.184	1.089	1.286	1.169	1.066	1.281
	400+	1.201	1.114	1.295	1.168	1.083	1.259	1.151	1.060	1.250
	Years 2 to 5:									
	<100	0.915	0.779	1.075	0.985	0.836	1.158	0.922	0.774	1.097
	400+	1.392	1.217	1.592	1.371	1.199	1.568	1.272	1.100	1.471
10 years	Years 0 to 2:									
	<100	1.071	0.986	1.163	1.186	1.092	1.289	1.169	1.067	1.282
	400+	1.201	1.114	1.295	1.167	1.082	1.258	1.154	1.063	1.254
	Years 2 to 10:									
	<100	0.909	0.789	1.046	0.973	0.845	1.121	0.912	0.782	1.064
	400+	1.379	1.225	1.552	1.365	1.213	1.536	1.324	1.164	1.506

1. Adjusted for age and sex.
2. Adjusted for age, sex, ethnicity, IMD quintile, smoking status, systolic blood pressure, total cholesterol, body mass index, prior history of angina, myocardial infarction, ischaemic heart disease, diabetes, hypertension, stroke, atrial fibrillation, valve disease and calendar period (2004-2010, 2011-2018)

Table S10: Association of BNP with all-cause mortality with BNP modelled as a continuous, linear exposure

		Unadjusted (N=13,529)			Partially adjusted[1] (N = 13,529)			Fully adjusted[2] (N = 11,913)		
Total follow-up	BNP value (Per 100 pg/ml)	HR	95% CI		HR	95% CI		HR	95% CI	
1 year		1.0047	1.0026	1.0069	1.0039	1.0017	1.0061	1.0033	1.0009	1.0057
5 years	Years 0 to 2	1.0055	1.0036	1.0073	1.0047	1.0028	1.0066	1.0041	1.0020	1.0062
	Years 2 to 5	1.0077	1.0042	1.0112	1.0078	1.0042	1.0115	1.0078	1.0038	1.0119
10 years	Years 0 to 2	1.0055	1.0036	1.0073	1.0047	1.0028	1.0066	1.0042	1.0021	1.0063
	Years 2 to 10	1.0083	1.0051	1.0115	1.0084	1.0051	1.0117	1.0088	1.0052	1.0124

1. Adjusted for age and sex.
2. Adjusted for age, sex, ethnicity, IMD quintile, smoking status, systolic blood pressure, total cholesterol, body mass index, prior history of angina, myocardial infarction, ischaemic heart disease, diabetes, hypertension, stroke, atrial fibrillation, valve disease and calendar period (2004-2010, 2011-2018)

Taylor CJ, *et al. Heart* 2022; 108:543–549. doi: 10.1136/heartjnl-2021-319196

Table S11: Association of NT-pro BNP and BNP with hospital admission for HF at 1 year

	Unadjusted (N=27,258)			Partially adjusted[1] (N = 27,258)			Fully adjusted[2] (N = 24,434)		
NT-pro BNP group (Reference =400-1999.9 pg/ml)	OR	95% CI		OR	95% CI		OR	95% CI	
<400	0.750	0.646	0.870	0.758	0.653	0.880	0.714	0.609	0.838
2000+	2.150	1.900	2.432	2.131	1.883	2.412	2.264	1.982	2.586
NT-pro BNP (per 100 pg/ml)	1.0068	1.0059	1.0067	1.0070	1.0057	1.0076	1.0071	1.0061	1.0082
	Unadjusted (N=13,529)			Partially adjusted[1] (N = 13,529)			Fully adjusted[2] (N = 11,913)		
BNP group (Reference = 100-399.9 pg/ml)	OR	95% CI		OR	95% CI		OR	95% CI	
<100	0.802	0.645	0.997	0.800	0.643	0.996	0.858	0.681	1.082
400+	1.849	1.562	2.188	1.842	1.556	2.180	1.817	1.514	2.181
BNP (per 100 pg/ml)	1.0127	1.0093	1.0161	1.0127	1.0093	1.0161	1.0123	1.0086	1.0161

1. Adjusted for age and sex.
2. Adjusted for age, sex, ethnicity, IMD quintile, smoking status, systolic blood pressure, total cholesterol, body mass index, prior history of angina, myocardial infarction, ischaemic heart disease, diabetes, hypertension, stroke, atrial fibrillation, valve disease and calendar period (2004-2010, 2010-2018).

Table S12: Association of NT-pro BNP with HF-related mortality at 1, 5 and 10 years of follow-up (estimated with competing risks models).

		Unadjusted (N=27,258)			Partially adjusted[1] (N = 27,258)			Fully adjusted[2] (N = 24,434)		
Total follow-up	NT-pro BNP value (Reference 400-1999.9 pg/ml)	HR	95% CI		HR	95% CI		HR	95% CI	
1 year	<400	0.739	0.588	0.930	0.870	0.691	1.096	0.804	0.623	1.038
	2000+	1.771	1.462	2.146	1.626	1.341	1.971	1.526	1.231	1.892
5 years										
	<400	0.747	0.593	0.939	0.878	0.697	1.105	0.812	0.629	1.048
	2000+	1.764	1.456	2.138	1.621	1.337	1.965	1.535	1.239	1.904
10 years										
	<400	0.745	0.592	0.937	0.877	0.696	1.104	0.811	0.629	1.047
	2000+	1.761	1.454	2.134	1.618	1.335	1.962	1.535	1.239	1.904

1. Adjusted for age and sex.
2. Adjusted for age, sex, ethnicity, IMD quintile, smoking status, systolic blood pressure, total cholesterol, body mass index, prior history of angina, myocardial infarction, ischaemic heart disease, diabetes, hypertension, stroke, atrial fibrillation, valve disease and calendar period (2004-2010, 2011-2018)

Taylor CJ, *et al. Heart* 2022; 108:543–549. doi: 10.1136/heartjnl-2021-319196

Figures

Figure S1: Flow diagram of included and excluded participants in CPRD Gold and Aurum databases

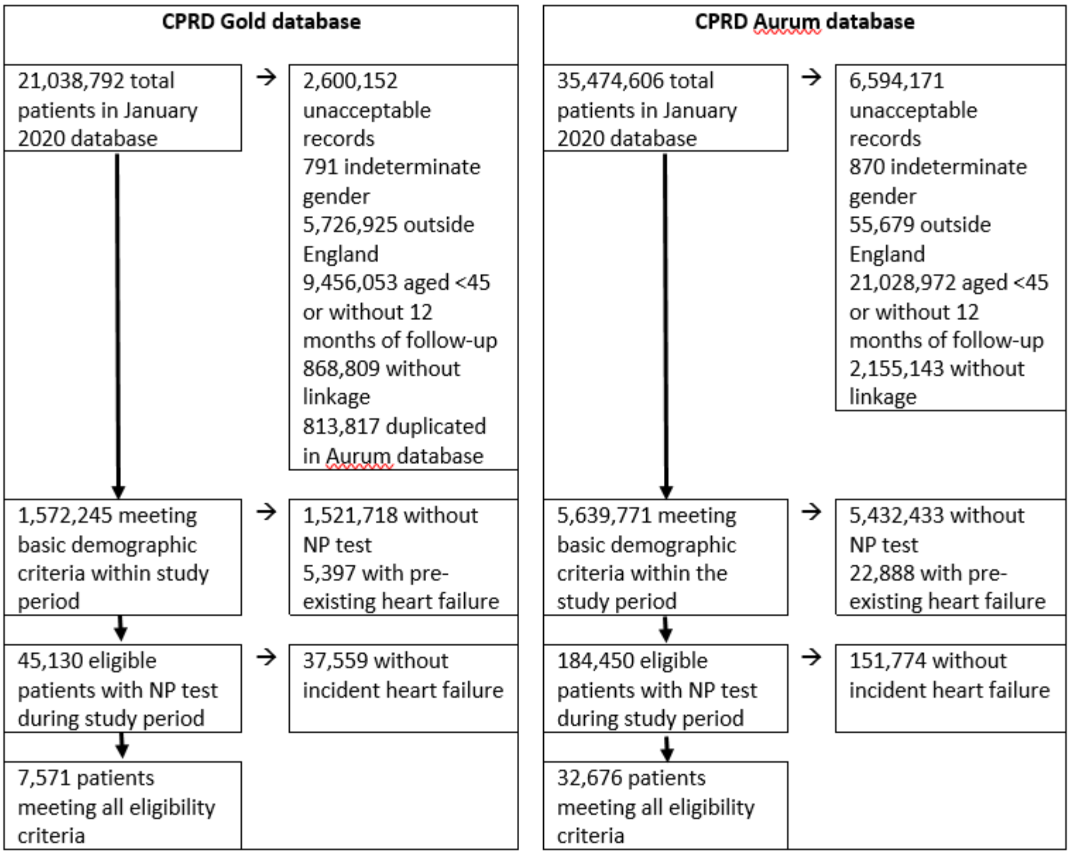

Taylor CJ, *et al. Heart* 2022; 108:543–549. doi: 10.1136/heartjnl-2021-319196

Figure S2: Distribution of BNP values at baseline

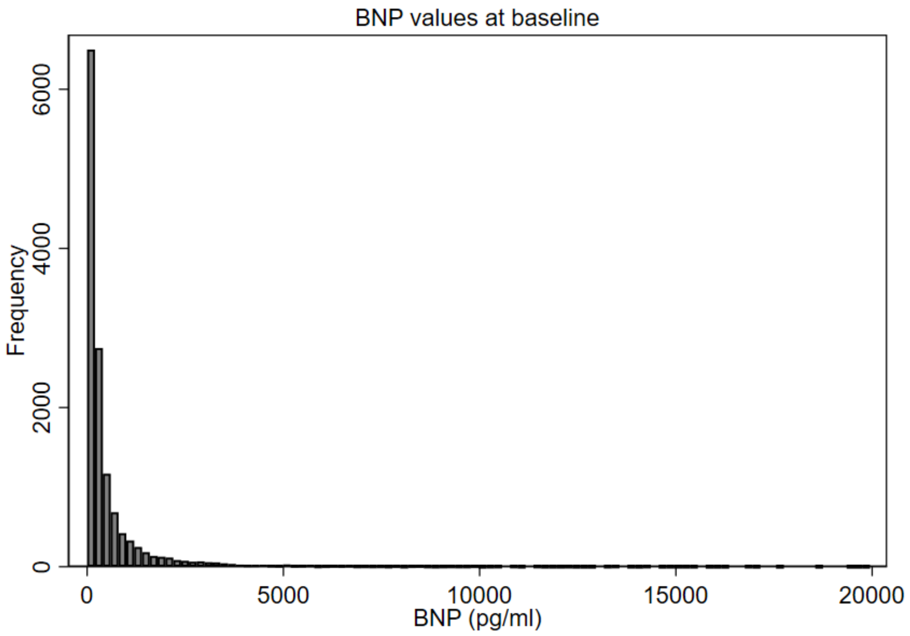

Figure S3: Distribution of NT-pro BNP values at baseline

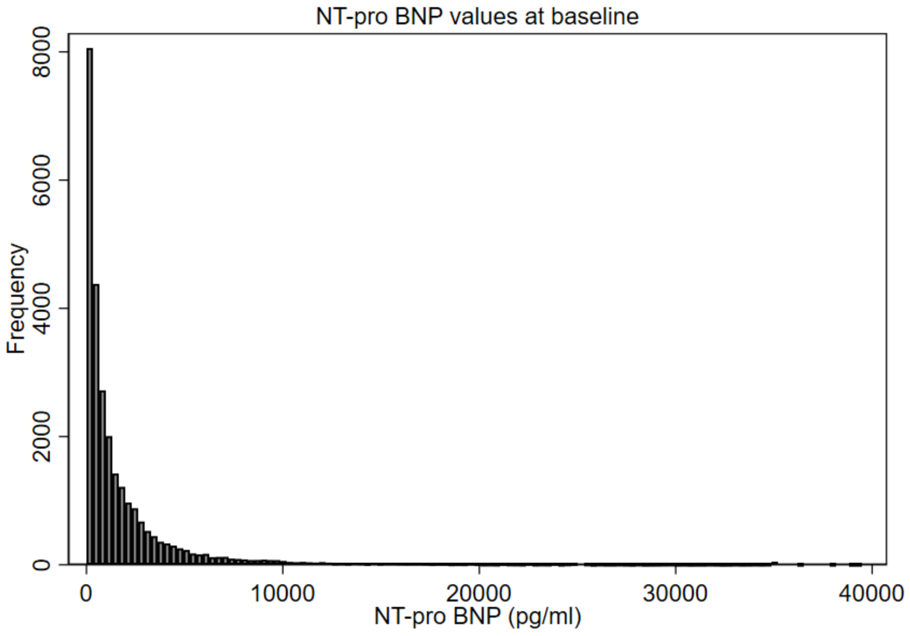

Taylor CJ, *et al. Heart* 2022; 108:543–549. doi: 10.1136/heartjnl-2021-319196

Figure S4: Survival by NT-pro BNP group (6 categories)

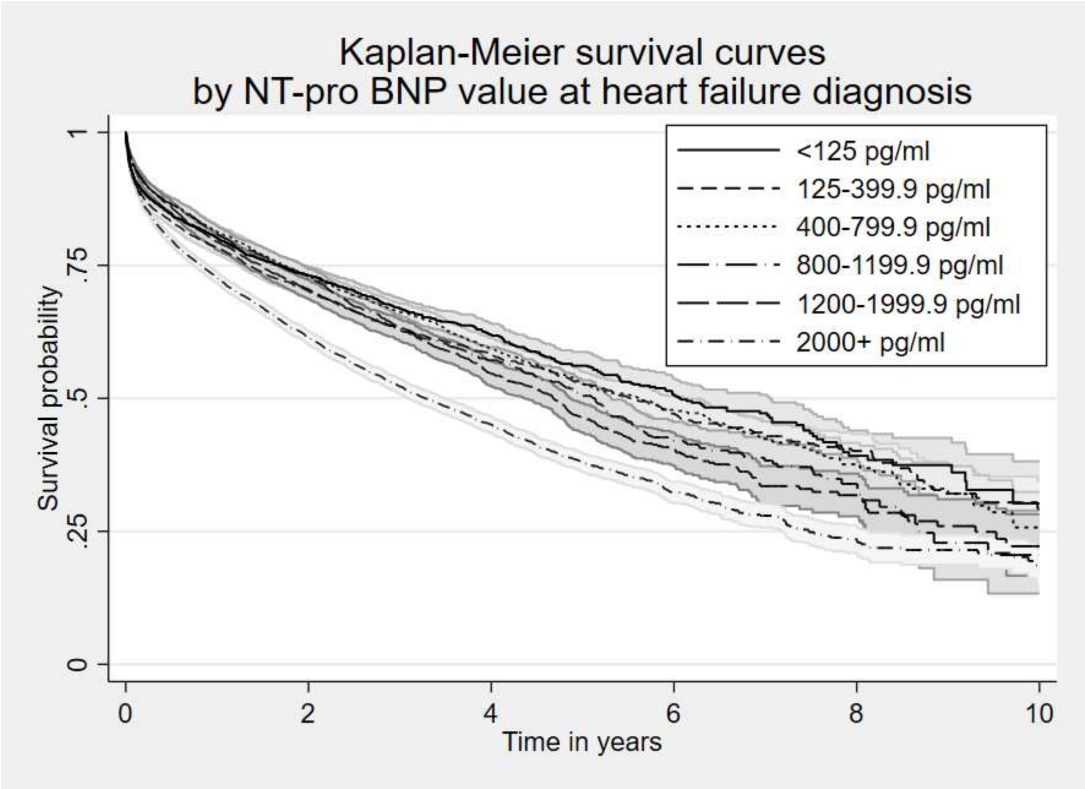

Taylor CJ, *et al. Heart* 2022; 108:543–549. doi: 10.1136/heartjnl-2021-319196
